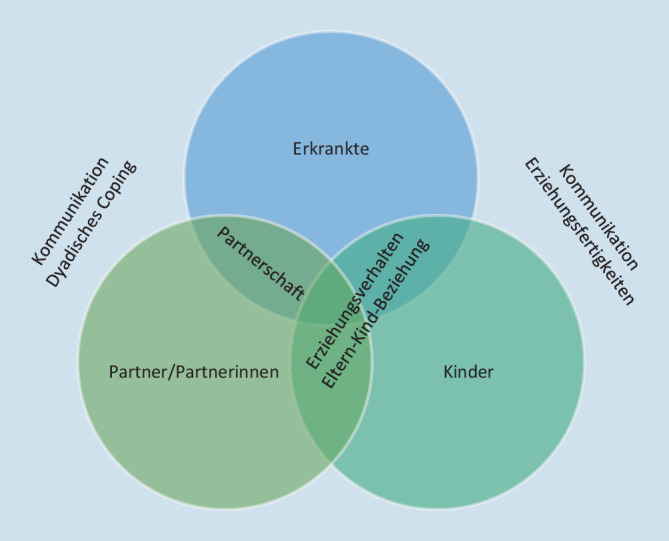# Partnerschaftliche und familiäre Aspekte bei Krebserkrankungen

**DOI:** 10.1007/s00103-022-03495-1

**Published:** 2022-02-18

**Authors:** Tanja Zimmermann

**Affiliations:** Klinik für Psychosomatik und Psychotherapie, Medizinische Hochschule Hannover, Carl-Neuberg-Str. 1, 30625 Hannover, Deutschland

**Keywords:** Cancer Survivorship, Langzeitüberleben nach Krebs, Partnerschaft, Familie, Paare, Cancer survivorship, Long-term survival after cancer, Partnership, Family, Couples

## Abstract

Eine Krebserkrankung stellt nicht nur für die erkrankte Person eine Herausforderung dar, sondern kann auch mit psychosozialen Beeinträchtigungen der Angehörigen, insbesondere der Partner und Partnerinnen und der minderjährigen Kinder, einhergehen. Obwohl Studien zu langfristigen Auswirkungen einer Krebserkrankung auf Partner und Partnerinnen, die Partnerschaft und die kindliche Entwicklung noch selten sind, gibt es Hinweise für die Chronifizierung der psychischen Belastungen von Angehörigen und für Risiken der Entwicklung psychischer Störungen bei Kindern erkrankter Eltern. Darüber hinaus erhöht eine Verschlechterung der partnerschaftlichen Zufriedenheit auch das Trennungsrisiko.

In der psychoonkologischen Versorgung sollten daher nicht nur die psychosozialen Belastungen der Erkrankten, sondern auch die der Partner und Partnerinnen und die Auswirkungen auf die Partnerschaft berücksichtigt werden und entsprechende Angebote z. B. zur Erhöhung der partnerschaftlichen und sexuellen Zufriedenheit, der Kommunikation oder dem dyadischen Coping erfolgen. Auch Kinder sollten in der Versorgung als Angehörige betrachtet werden. Ein Fokussieren auf die kindlichen Belastungen sowie auf die Verbesserung der Eltern-Kind-Beziehung und des Erziehungsverhaltens kann Kindern bei der Bewältigung der elterlichen Erkrankung auch langfristig helfen. Somit sollte eine Krebserkrankung immer auf individueller, dyadischer und familiärer Ebene betrachtet werden und psychosoziale Versorgungsangebote sollten auf allen Ebenen erfolgen.

## Abstract (en)

Cancer is not only a challenge for the patient, but can also be associated with psychosocial distress for relatives, especially partners and minor children. Although studies on the long-term effects of cancer on partners, the partnership, and child development are still rare, there are indications that the psychological distress on relatives may become chronic, and that parental illness is a risk factor for a child to develop mental disorders. In addition, a deterioration in partnership satisfaction also increases the risk of separation.

Psycho-oncology care should therefore not only consider the patient’s psychosocial burden but also that of the partner, as well as its effect on the partnership. Appropriate offers, for example to increase partnership and sexual satisfaction, communication, or dyadic coping, should be made. Children should also be treated as relatives in care. Focusing on both the child’s distress and on improving the parent-child relationships and parenting behavior can help children to cope with parental illness in the long term. Thus, cancer should be viewed on the individual, dyadic, and family level, and psychosocial care services should be provided at all levels.

## Einleitung

In Deutschland wird im Laufe des Lebens jede zweite Person an Krebs erkranken. Trotz steigender Inzidenzen findet sich aufgrund der verbesserten Krebsfrüherkennung und -therapie eine sinkende Mortalität. Die Zahl der Krebsüberlebenden (*Cancer Survivors*) wächst in den letzten Jahrzehnten zunehmend und liegt in Deutschland aktuell bei ca. 4 Mio. Menschen [1]. Als *Cancer Survivor* gelten entsprechend der Definition der National Coalition for Cancer Survivorship (NCCS) Personen, die in ihrem Leben mit einer Krebserkrankung konfrontiert wurden – unabhängig von Prognose, Krankheitsverlauf und -status [2]. Eine Krebserkrankung kann mit medizinischen, sozialen, emotionalen und existenziellen Belastungen für die erkrankte Person einhergehen, die auch nach dem Ende der Behandlung andauern und das Überleben beeinflussen können. Das mittlere Erkrankungsalter für Krebs liegt bei Ende 60 Jahren. In diesem Alter leben über 60 % der Menschen in einer Partnerschaft. Eine Krebserkrankung wirkt sich somit nicht nur auf die erkrankte Person aus, sondern auch auf das Leben der Angehörigen – in erster Linie auf die Partner und Partnerinnen sowie ggf. die Kinder. Lag der Fokus der Forschung lange Zeit auf den psychosozialen Auswirkungen einer Krebserkrankung auf die erkrankte Person, haben sich Studien in den letzten Jahren zunehmend mit den Auswirkungen auf die Partner und Partnerinnen [3] sowie minderjährige Kinder [4] beschäftigt [5]. Studien zum *längsschnittlichen Verlauf *psychosozialer Belastungen bei Erkrankten, Partnern und Partnerinnen sowie Kindern sind jedoch noch selten ebenso wie zu Belastungen beim erweiterten familiären Umfeld, wie den eigenen Eltern oder erwachsenen Kindern. Im Folgenden sollen die psychosozialen Auswirkungen einer Krebserkrankung auf Partner und Partnerinnen, die Partnerschaft und minderjährige Kinder genauer beleuchtet werden.

## Auswirkungen einer Krebserkrankung auf Partner und Partnerinnen

Eine Krebserkrankung stellt einen *Stressor* dar, der nicht nur die erkrankte Person, sondern auch die Partner und Partnerinnen belasten kann. Auch diese erleben psychische Belastungen wie Ängste, Hilf- und Hoffnungslosigkeit oder Stimmungsbeeinträchtigungen sowie eine eingeschränkte Lebensqualität [6–9]. Darüber hinaus können ein hoher *Distress* und eine geringe Partnerschaftszufriedenheit auch eine Belastung für die Beziehung darstellen [10, 11]. Insbesondere die Partner und Partnerinnen sind mit den dauerhaften Krankheitsfolgen wie Rollenveränderungen, Belastungen, Krankheitsverarbeitung oder Interaktionsprozessen konfrontiert [12]. Sie befinden sich oft in einer Doppelrolle: Einerseits sind sie für die Erkrankten die wichtigste Quelle für emotionale und praktische Unterstützung [13, 14], andererseits erleben sie eigene psychosoziale Belastungen, die Unterstützung erforderlich machen [15]. Sie befinden sich somit in einem Spannungsfeld zwischen den Erwartungen der erkrankten Person, des sozialen Umfelds und Behandlungsteams sowie den eigenen Belastungen, der Ohnmacht und Hilflosigkeit. Obwohl viele Partner und Partnerinnen ihre Rolle auch als positiv und bedeutsam erleben [16], weisen insbesondere pflegende Partner und Partnerinnen ein hohes Ausmaß an Depressivität und Ängstlichkeit sowie ein höheres Risiko für eigene Erkrankungen und eine höhere Mortalität im Vergleich zu nicht pflegenden Partnern und Partnerinnen auf [17]. Für die Anpassung der Partner und Partnerinnen an die Erkrankung spielen auch medizinische Faktoren wie Verlauf und Prognose, Behandlungsart und Spätfolgen, aber auch die eigene psychische Labilität und somatische Beeinträchtigungen eine wichtige Rolle [18].

In den letzten Jahren wurden diese Belastungen für Partner und Partnerinnen zunehmend separat betrachtet, beziehen sich jedoch meist auf die Krankheitsphase der Diagnosevermittlung, der akuten medizinischen Behandlung oder die Palliativ- bzw. Terminalphase [15]. Auch wenn Studien zur Langzeitbelastung der Partner und Partnerinnen noch eher selten sind, weisen erste Ergebnisse darauf hin, dass ihre psychische Belastung 5 Jahre nach der Diagnosestellung nicht höher ist als in der Allgemeinbevölkerung [19].

Dennoch zeigen ca. 30 % der Partner und Partnerinnen psychosozialen Distress [15], wobei Frauen als Angehörige stärker betroffen sind als Männer [20]. Psychische Belastung geht häufig mit einer geringeren Partnerschaftszufriedenheit einher und beeinträchtigt somit auch die Erkrankten sowie die gegenseitige Unterstützung [7]. Es zeigt sich zudem, dass Partner und Partnerinnen trotz mindestens vergleichbarer psychosozialer Belastung weniger Unterstützung – sowohl vom sozialen Umfeld als auch vom medizinischen Team – erhalten als die Erkrankten [21]. Auch wenn bisher nur wenige Forschungsergebnisse zum Verlauf der Belastungen sowie den Folgen im Längsschnitt vorliegen, weisen erste Studien darauf hin, dass sich die psychische Belastung der Partner und Partnerinnen *chronifizieren* kann.

## Auswirkungen auf die Partnerschaft

Eine Krebserkrankung kann mit vielfältigen Veränderungen in der Partnerschaft einhergehen. Oft kommt es zu Rollenveränderungen mit möglicherweise auch pflegerischen Aufgaben der Partner und Partnerinnen. Insbesondere die *Asymmetrie* in der Partnerschaft mit einer Zuschreibung der Gesunden- und Krankenrolle kann im langfristigen Verlauf eine Herausforderung darstellen. Sich aus dieser Asymmetrie wieder zu befreien, gelingt nicht immer. Zudem kann die Kommunikation bei Paaren beeinträchtigt sein: Vielen Betroffenen fällt es schwer, über Ängste und Sorgen hinsichtlich der Krebserkrankung zu sprechen. Dies führt häufig dazu, dass eine Person ihre eigenen Ängste und Sorgen vor der anderen Person verbirgt, um diese nicht zusätzlich zu belasten. Dieses als *Protective Buffering* bezeichnete Phänomen geht mit einer schlechteren Anpassung der Paare an die Herausforderungen der Krebserkrankung einher [22].

Seit den 1990er-Jahren werden *Stress und Coping *als interpersonelle Prozesse betrachtet. Somit sollte die Krebserkrankung auch als Stressor für beide Personen gleichzeitig und die Partnerschaft betrachtet werden. Der Begriff der *Wir-Erkrankung (We-Disease)* hat sich in diesem Zusammenhang geprägt [3]. Beide Personen können durch medizinische, psychosoziale oder existenzielle Stressoren belastet sein. Beide verfügen aber auch über Ressourcen, die bei der Bewältigung hilfreich sein können. Für die meisten Erkrankten stellen die Partner und Partnerinnen die wichtigste Unterstützungsressource dar [14, 23]. Insbesondere persönliche und soziale Ressourcen sind von großer Bedeutung bei Cancer Survivors. In einer Studie mit 6030 langzeitüberlebenden Brust‑, Darm- und Prostatakrebserkrankten erwiesen sich die Familie, Aktivitäten mit anderen und die Partnerschaft als hilfreiche Ressourcen [24].

Dennoch kann auch die psychische Belastung der Partner und Partnerinnen negative Auswirkungen auf die Partnerschaft haben. Eine hohe Partnerschaftszufriedenheit ist ein wichtiger Schutzfaktor für eine bessere Gesundheit [25], wohingegen eine Unzufriedenheit in der Partnerschaft einen Hochrisikofaktor für psychische oder physische Störungen darstellt. Tatsächlich hat die partnerschaftliche Beziehung einen positiven Einfluss auf den Zeitpunkt der Diagnose (früheres Erkrankungsstadium), das Behandlungsergebnis und die Krebssterblichkeit. Demnach ist es von Vorteil, wenn sich Krebserkrankte in einer Partnerschaft befinden [26]. Buja et al. [27] zeigten in ihrer systematischen Übersichtsarbeit, dass unverheiratete Patienten und Patientinnen ein höheres Risiko für fortgeschrittenen Krebs oder Melanom zum Zeitpunkt der Diagnose haben. Darüber hinaus wiesen Unverheiratete ein höheres Risiko für metastasierten Krebs, Unterbehandlung sowie eine höhere Mortalität als verheiratete Erkrankte auf [28]. Mit der sozialen Unterstützung ihrer Partner und Partnerinnen sind Erkrankte eher in der Lage, die Herausforderungen einer Krebsdiagnose, einschließlich etwaiger Depressionen, Ängste, sowie berufliche und finanzielle Probleme zu bewältigen und einen gesunden Lebensstil und eine positive Einstellung aufrechtzuerhalten [29]. Partner und Partnerinnen scheinen Erkrankten somit eine emotionale und praktische Unterstützung zu bieten, die Behandlungsentscheidungen erleichtern und positive Effekte auf das Gesundheitsverhalten und die Rehabilitation zeigen kann [30].

In den meisten Fällen finden Paare Wege, mit den herausfordernden Stressoren einer Krebserkrankung gemeinsam umzugehen. Sie beschreiben auch positive Auswirkungen der Erkrankung auf die Partnerschaft, z. B. dass sie sich im Sinne einer *Kohäsion* einander näher fühlen, sich aufeinander verlassen können und ein gutes Team sind [30]. Wenn jedoch die dyadische Anpassung (*dyadisches Coping*) an krebsbedingte Stressoren nicht gelingt, kann die Stabilität der Beziehung bedroht sein [31]. Einige Studien zeigen ein erhöhtes Risiko für Trennung oder Scheidung von Paaren mit Krebserkrankungen im Vergleich zur Allgemeinbevölkerung [32, 33]. Andere Studien wiederum fanden kein größeres Trennungs- oder Scheidungsrisiko für Krebsüberlebende [34]. Wenige Studien beschreiben Geschlechterunterschiede, bei denen Patientinnen häufiger eine Trennung oder Scheidung erleben als Patienten [32, 35, 36]. In einer aktuellen Studie aus Deutschland wurden 265 Partner und Partnerinnen von Krebserkrankten hinsichtlich einer möglichen Trennung, der Trennungsgründe sowie des Einflusses der Krebserkrankung auf die Partnerschaft befragt. Die Ergebnisse zeigten eine deutlich geringere Trennungsrate als in der deutschen Allgemeinbevölkerung. Im Falle einer Trennung werden als häufigste Gründe Partnerschaftsprobleme und die Krebserkrankung genannt: Über die Hälfte der getrennten Paare berichteten, dass der Krebs zur Trennung beigetragen habe. Insgesamt berichteten über 80 % der Befragten, dass die Krebserkrankung die Beziehung beeinflusst hat. Circa 56 % beschrieben dabei einen negativen Einfluss. Ein höheres Trennungsrisiko sowie eine negative Wahrnehmung des Einflusses von Krebs auf die Partnerschaft waren mit höherer Depressivität der Partner und Partnerinnen verknüpft, wohingegen eine höhere Partnerschaftszufriedenheit mit einer positiven Wahrnehmung einherging [11]. Die Ergebnisse dieser Studie bestätigen, dass die Partner und Partnerinnen auch langfristig unter den Konsequenzen einer Krebserkrankung leiden können und die besondere Herausforderung in der langfristigen Anpassung an die veränderten Lebensumstände liegt. Dazu gehören z. B. dauerhafte Einschränkungen in der Funktionsfähigkeit (z. B. auch im Bereich der Sexualität), veränderte Rollen, Progredienzängste, abweichende Lebens- oder Zukunftspläne [30].

Da eine zufriedene Partnerschaft einen wichtiger Schutzfaktor sowohl für die Erkrankten als auch für die Partner und Partnerinnen darstellt, ist der Einbezug der Partner und Partnerinnen in die psychoonkologische Versorgung hinsichtlich der Aufrechterhaltung einer zufriedenen Partnerschaft, aber auch der Verbesserung der partnerschaftlichen Kommunikation und Stressbewältigung sowie der Reduktion der psychischen Belastung beider sinnvoll [37]. Auch nach Abschluss der medizinischen Behandlung können die Auswirkungen der Krebserkrankung auf die gesamte Lebens- und Zukunftsplanung (z. B. Familienplanung, berufliche Entwicklung) noch eine große Herausforderung für Paare darstellen und mit chronischem Stress assoziiert sein, der die Partnerschaft belastet. Chronische Stressbelastungen in einer Partnerschaft führen zu einer Verschlechterung der Kommunikation, Verringerung der Intimität und des emotionalen Wohlbefindens [38]. Darüber hinaus findet sich eine *Interdependenz* sowohl für Belastungen als auch für Bewältigungsstrategien [39]. Daher hat sich das dyadische Coping, d. h. der gemeinsame Umgang des Paares mit diesen Stressoren, als eine wichtige Ressource für die Anpassung an die Krebserkrankung erwiesen [40]. Das dyadische Coping beschreibt das Zusammenspiel zwischen den Stresssignalen einer Person, der Wahrnehmung dieser Signale durch die andere Person und dem gemeinsamen Umgang damit [41]. Hierfür ist die partnerschaftliche Kommunikation essenziell: Beide Personen müssen in der Lage sein, ihren empfundenen Stress angemessen zu äußern sowie die Stresssignale des anderen wahrzunehmen und darauf angemessen zu reagieren [40]. Ein erfolgreiches dyadisches Coping trägt zu einer Steigerung des Wir-Gefühls bei und geht mit einer höheren Partnerschaftszufriedenheit und einem höheren Kohäsionsgefühl einher [42].

Eine Krebserkrankung kann häufig auch zu sexuellen Funktionsstörungen führen, welche wiederum die partnerschaftliche Zufriedenheit beeinträchtigen können [43]. Die Ursachen sind vielfältig und können aus Veränderungen oder Beeinträchtigungen des Körperbildes oder der Körperfunktionen resultieren sowie dem partnerschaftlichen oder dem psychosozialen Erleben. Veränderungen des Körperbildes (wie Narben, Verlust von Körperteilen, Gewichtsveränderungen, Haarverlust etc.) oder der Körperfunktionen (z. B. Inkontinenz, Impotenz, Verlust der Stimme, Fatigue), die durch medizinische Behandlungen ausgelöst sein können, beeinträchtigen mitunter das Selbstwertgefühl, das Bedürfnis nach sexueller Aktivität und die Libido. Darüber hinaus können sie auch depressive Symptome, Angst und Insuffizienzgefühle begünstigen [44]. Insbesondere die Veränderungen in den partnerschaftlichen Rollen – z. B. von der pflegenden Rolle wieder in die Rolle als Liebende oder Liebender zu wechseln – können für einige Paare eine große Herausforderung darstellen, die möglicherweise auch langfristig zu Schwierigkeiten führt. Hinzu kommt, dass es vielen Paaren schwerfällt, offen über Sexualität zu sprechen und Ängste sowie Sorgen diesbezüglich häufig nicht adressiert werden. Dies wiederum kann zu Missverständnissen und Unzufriedenheit führen. Hinzu kommen oft Wissensdefizite und Missverständnisse, die aus einer fehlenden Aufklärung durch Fachleute über Nebenwirkungen der onkologischen Behandlung auf die sexuelle Funktionsfähigkeit resultieren. Die Ursachen für sexuelle Funktionsstörungen werden dann bei sich selbst oder der anderen Person gesucht, ohne dies aber offen zu kommunizieren. So kann sich die Kommunikationsstörung diesbezüglich bis zur Sprachlosigkeit ausweiten und zur Folge haben, dass Paare nach der Krebsdiagnose auf jede Form von Zärtlichkeit, Körperkontakt und Sexualität verzichten [45]. Ein offener Umgang – sowohl zwischen Behandelnden und Erkrankten als auch in der Partnerschaft – kann die Wiederaufnahme sexueller Aktivitäten begünstigen und somit eine zufriedenstellende Sexualität nach einer Krebserkrankung ermöglichen.

Die Ergebnisse bisheriger Studien betonen deutlich die Wichtigkeit des Einbezugs der Partner und Partnerinnen in die psychoonkologische Versorgung sowie die Berücksichtigung partnerschaftlicher Aspekte wie Kommunikation, partnerschaftliche Unterstützung (dyadisches Coping) und Sexualität.

## Auswirkungen auf minderjährige Kinder und die Familie

Eine weitere in der klinischen Versorgung häufig noch vernachlässigte Gruppe von Angehörigen stellen die minderjährigen Kinder Krebserkrankter dar. Für Kinder sind die Eltern während der gesamten Entwicklung und insbesondere hinsichtlich der Verhaltens- und Emotionsregulation die wichtigsten Bezugspersonen und die Eltern-Kind-Beziehung hat einen bedeutenden Einfluss auf eine gesunde Entwicklung von Kindern und Jugendlichen. Erkrankt ein Elternteil körperlich oder psychisch, kann dies auch einen Risikofaktor für die kindliche Entwicklung darstellen [46]. Zum Einfluss einer elterlichen Erkrankung auf minderjährige Kinder gibt es erst seit einigen Jahren Studien, die zeigen, dass die elterliche Erkrankung erhebliche Auswirkungen auf das Familienleben und auch die kindliche Entwicklung haben kann [47, 48]. In bisherigen Studien zeigen die meisten Kinder keine psychischen Auffälligkeiten. Allerdings scheinen Alter und Geschlecht (jugendliche Mädchen, jüngere Jungen und jüngere Eltern) Einflussfaktoren auf die kindliche Belastung zu sein [48, 49]. In einer finnischen Kohortenstudie fanden sich keine höheren Prävalenzen für psychische Störungen bei Kindern krebskranker Eltern, auch wenn sich hier geschlechtsspezifische Risikofaktoren zeigten [50], wohingegen eine Registerstudie aus Schweden [51] sowie eine populationsbasierte Studie aus Dänemark [52] ein höheres Risiko für psychische Störungen bei Kindern aufzeigen.

Die elterliche Erkrankung stellt einen Risikofaktor für die kindliche Entwicklung und das Auftreten späterer psychischer Auffälligkeiten oder Erkrankungen dar [4]. Darüber hinaus können behandlungsbedürftige Symptome erst nach mehrjähriger Latenz anscheinender Symptomfreiheit entstehen. Möglicherweise werden die Beschwerden von den Eltern auch unterschätzt, da sie sich selber in einer emotionalen Belastungssituation befinden und das Verhalten des Kindes nicht genau einschätzen können [53, 54]. Mögliche Probleme sind, dass Kinder die Angst und Verunsicherung ihrer Eltern spüren und dann versuchen, möglichst stark zu sein, und so in ihren Emotionen missverstanden werden. In einer aktuellen Studie aus den USA zeigten 25 % der Kinder krebskranker Eltern ein hohes Level an Distress sowie eine beeinträchtigte Lebensqualität [55].

Die Erkrankung eines Elternteils hat auch erhebliche Auswirkungen auf die familiären Lebens- oder Zukunftspläne, Abläufe und Rollenzuteilungen und kann Unsicherheiten und Ängste bei den Kindern auslösen [48]. Eine Übersichtsarbeit reflektiert den Einfluss der elterlichen Erkrankung auf den Alltag der Kinder (Unterbrechungen in der täglichen Routine), Rollenveränderungen sowie emotionale Probleme und soziale Funktionsfähigkeit der Kinder [56].

Wenn ein Elternteil an Krebs erkrankt, findet sich auch im familiären Zusammenhalt ähnlich wie bei Paaren zunächst eine *Kohäsion*, d. h., das Familienbindungssystem, welches Sicherheit, Halt, Trost und Orientierung bietet, wird durch die elterliche Erkrankung aktiviert [48]. Allerdings kann eine extrem hohe Kohäsion auch zu einer Überidentifizierung mit der Familie führen und eine extrem schwache Bindung zu einer Loslösung der Familienmitglieder. Demzufolge ist ein mittleres Ausmaß an Kohäsion und Einbindung als funktional und adaptiv zu betrachten [48].

In vielen Familien kommt es zu einer sozialen Isolation, da die Anforderung der Erkrankung die Familienmitglieder dermaßen beansprucht, dass die Pflege sozialer Außenkontakte darunter leidet. Möglicherweise wird die Krebserkrankung als *familieninterne *Angelegenheit betrachtet und somit nicht offen mit anderen darüber gesprochen. Bei Kindern führt dies z. B. dazu, dass sie es vermeiden, Freunde einzuladen oder mit anderen über die Erkrankung des Elternteils zu sprechen [48]. Die Flexibilität in der Familie kann eingeschränkt werden, d. h., Aktivitäten sind beispielsweise nicht mehr so spontan möglich oder benötigen eine lange Vorausplanung. Des Weiteren werden Konflikte in der Familie häufig vermieden beziehungsweise aus Rücksichtnahme nicht mehr angesprochen [48]. Durch die Erkrankung eines Elternteils müssen Kinder häufig mehr Aufgaben z. B. im Haushalt oder bei der Versorgung der jüngeren Geschwister übernehmen. Dies kann ihnen einerseits bei der Bewältigung der eigenen Hilflosigkeit helfen, andererseits müssen diese Aufgaben altersangemessen sein, da sie sonst eine Überforderung (*Parentifizierung*) für das Kind darstellen [48]. Zudem ist darauf zu achten, dass Kinder keine emotionale Partnerersatzrolle übernehmen.

Eltern fühlen sich oft unsicher hinsichtlich der Aufklärung der Kinder über die Krebserkrankung und sind möglicherweise durch deren sehr direkte Fragen und Verhaltensweisen überfordert und hilflos [47]. Kinder können sehr unterschiedliche Emotionen zeigen, wie z. B. Angst vor dem Verlust des Elternteils, Wut gegenüber dem Krebs oder dem erkrankten Elternteil, Schuldgefühle (wenn sie sich verantwortlich für die Krebsentstehung fühlen) oder aber auch Neugier, die sich durch direkte Fragen zur Behandlung oder den Nebenwirkungen äußert [57]. Darüber hinaus kann die elterliche Erkrankung auch Veränderungen der Stimmung, des Sozialverhaltens, regressive Symptome, Änderungen des Schulverhaltens, antisoziales Verhalten, gesteigertes Gefühl der eigenen Verantwortung etc. zur Folge haben. Eine Herausforderung in der klinischen Versorgung sind die Identifikation von Familien und Kindern mit einer *dysfunktionalen Funktionsfähigkeit* und das Angebot entsprechender psychoonkologischer Versorgungsangebote.

Im Umgang mit der elterlichen Erkrankung hat es sich als hilfreich erwiesen, wenn die Familie in der Lage ist, eine gemeinsame Problemsicht und -definition zu entwickeln. Gute Copingfertigkeiten und eine hohes familiäres Funktionsniveau haben sich als Prädiktoren für das psychische Wohlbefinden der Kinder herausgestellt [49]. Darüber hinaus sind starke, tragfähige emotionale Bindungen innerhalb der Familie ebenso wie ein optimistisches Familienselbstbild („Wir halten zusammen, sind ein Team“) hilfreich. Auch bewährte Prinzipien zum Umgang mit Stressbelastungen sowie die Bereitschaft, offen über die Erkrankung – auch im sozialen Umfeld – zu sprechen, sind günstige Strategien zum Umgang mit der Krebserkrankung eines Elternteils [57]. Resiliente Kinder weisen darüber hinaus, trotz schwieriger Lebensumstände, eine stabile und emotional sichere Bindung zu mindestens einer Bezugsperson auf, die eine fürsorgliche Betreuung und Förderung leistet. Diese Bezugsperson kann neben dem erkrankten Elternteil auch das gesunde Elternteil oder andere Personen wie Großeltern oder nahe Verwandte sein [58]. Eine besondere Bedeutung hat hierbei auch die elterliche Kommunikation [54]. Eltern sind möglicherweise verunsichert, ob überhaupt und wie sie Kinder über die Krebserkrankung informieren sollten. Eltern zu ermutigen, offen und ehrlich über die Erkrankung und die direkten Folgen für die Kinder zu sprechen, kann auch Kindern helfen [59].

Neben Veränderungen der familiären Situation und Abläufe beeinflusst die Erkrankung in vielen Fällen auch das Erziehungsverhalten der Eltern [60, 61]. Dieses kann entweder sehr rigide, eher vernachlässigend oder übermäßig verwöhnend und überbehütend werden. Allerdings stellt eine inkonsistente Erziehung ein Risiko für die weitere sozioemotionale Entwicklung des Kindes dar und kann langfristig eine Verschlechterung der Eltern-Kind-Beziehung sowie Auswirkungen auf die psychische Entwicklung des Kindes zur Folge haben [62]. Insbesondere in Zeiten von Angst und erhöhtem Stress durch eine Krebserkrankung ist eine verlässliche Eltern-Kind-Beziehung für die Kinder sehr wichtig. Die beste Hilfe für Kinder krebskranker Eltern ist, wenn die Eltern trotz der subjektiven Instabilität eine *objektive Stabilität* innerhalb der Familie aufrechterhalten. Idealerweise sollten beide Elternteile zusammenarbeiten und gemeinsame Ziele im Umgang mit der Erkrankung verfolgen. Kinder sollten trotz der Erkrankung weiterhin das Leben eines Kindes führen können und nicht das eines Erwachsenen. Die von den Kindern benötigte Sicherheit können Eltern unabhängig vom Erkrankungsstadium immer geben, denn Sicherheit entsteht durch positive Kommunikation, klare Strukturen, positive Aufmerksamkeit und ein liebevolles Miteinander. In diesem Zusammenhang ist die Stärkung der Eltern-Kind-Beziehung von größter Bedeutung.

*Elterntrainings* bieten eine hervorragende Möglichkeit, die Beziehung von Eltern und Kind positiv zu gestalten, die Entwicklung der Kinder zu fördern sowie internalisierende und/oder externalisierende Auffälligkeiten der Kinder zu reduzieren [63]. Zudem reduzieren sie die alltägliche Belastung der Eltern. Die beste empirische Evidenz weisen Elterntrainings auf, die auf den Aufbau einer positiven Beziehung zum Kind fokussieren. Hierzu sind 2 wichtige Strategien zu nennen: Zuneigung zeigen (körperlich) und verbaler Umgang (mit dem Kind reden, Interesse zeigen; [64]). Während der akuten Behandlung kann das Thema Erziehung auch an Priorität verlieren, welches mittel- und langfristig für die kindliche Entwicklung jedoch ungünstig ist. Ungünstige Erziehungsstrategien führen auch zu einer größeren Stressbelastung der Eltern [61]. Daher scheint es sinnvoll, Eltern mit einer Krebserkrankung das Thema Erziehung niedrigschwellig und leicht zugänglich anzubieten – besonders im Hinblick auf die zeitgleiche Belastung durch die Krebserkrankung.

## Fazit

Eine Krebserkrankung geht nicht nur mit psychosozialen Belastungen für die erkrankte Person einher, sondern wirkt sich auch auf die Partner und Partnerinnen und die Partnerschaft sowie die gesamte Familie aus. Das Vorhandensein einer Partnerschaft stellt einen gesundheitsfördernden Faktor dar und viele Partner und Partnerinnen erleben ihre Rolle als bereichernd und positiv. Dennoch verändert die Erkrankung bei vielen Paaren den partnerschaftlichen Alltag und die psychosoziale Funktionsfähigkeit deutlich. Das dyadische Coping hat sich als wirkungsvoll erwiesen, um mit diesen Belastungen umzugehen und zu einer Steigerung des Wir-Gefühls beizutragen, welches die Zufriedenheit mit der Partnerschaft erhöht. Auch die Auswirkungen der Krebserkrankung und -behandlung auf die sexuelle Funktionsfähigkeit sind bei Paaren zu berücksichtigen: Neben medizinischen, psychosozialen und existenziellen Stressoren erleben viele Paare sexuelle Probleme, die entweder aus der medizinischen Behandlung oder aber aus den psychischen Belastungen oder Körperbildstörungen resultieren können. Diese Probleme können zu Angst, Depressivität, Insuffizienzgefühlen und Isolation sowie zu einer Sprachlosigkeit über das Thema Sexualität – sowohl in der Partnerschaft als auch mit dem medizinischen Team – führen. Zur Bewältigung der Herausforderung einer Krebserkrankung haben sich im Umgang mit Paaren insbesondere Kommunikationsfertigkeiten, wie sie z. B. im Rahmen von paarbasierten Unterstützungsangeboten vermittelt werden, als hilfreich erwiesen.

Auch Kinder können Belastungen durch die elterliche Krebserkrankung erleben. Daher ist es für die psychoonkologische Versorgung wichtig, zum einen Eltern mit einer Krebserkrankung in ihrer Elternrolle zu stärken, zum anderen aber auch die Bedürfnisse der Kinder besser wahrzunehmen. Hier kann es hilfreich sein, Eltern darin zu stärken, eine offene Kommunikation über die Erkrankung zu gewährleisten. Dies erleichtert es Kindern, Fragen zu stellen und eigene Ängste oder Fantasien offen anzusprechen. Werden Kinder aktiv mit einbezogen – allerdings ohne sie dabei zu überfordern –, haben sie die Gelegenheit, aktive Bewältigungsstrategien zu entwickeln und anzuwenden.

Die behandelnden Fachpersonen sollten daher neben den Erkrankten stets auch die Angehörigen – sowohl die Partner und Partnerinnen als auch Kinder – im Blick haben. Die Vermittlung und Förderung individueller, dyadischer und familiärer Copingstrategien kann sowohl den Erkrankten als auch den Partnern und Partnerinnen und Kindern sowie der Familie als Ganzes in dieser herausfordernden Zeit und darüber hinaus helfen (Abb. 1).

**Figure 1:**